# Effect of nutrition in Alzheimer’s disease: A systematic review

**DOI:** 10.3389/fnins.2023.1147177

**Published:** 2023-05-04

**Authors:** Inmaculada Xu Lou, Kamran Ali, Qilan Chen

**Affiliations:** ^1^International Education College of Zhejiang Chinese Medical University, Hangzhou, Zhejiang, China; ^2^Department of Cardiology, Hangzhou Hospital of Traditional Chinese Medicine, Hangzhou, Zhejiang, China; ^3^Department of Oncology, The Fourth Affiliated Hospital, International Institutes of Medicine, Zhejiang University School of Medicine, Hangzhou, Zhejiang, China

**Keywords:** Alzheimer’s disease, nutrition, microbiota, Western diet, Mediterranean diet

## Abstract

**Background and objective:**

Alzheimer’s disease (AD) is a progressive neurodegenerative disease characterized by declining cognitive ability. Currently, there are no effective treatments for this condition. However, certain measures, such as nutritional interventions, can slow disease progression. Therefore, the objective of this systematic review was to identify and map the updates of the last 5 years regarding the nutritional status and nutritional interventions associated with AD patients.

**Study design:**

A systematic review.

**Methods:**

A search was conducted for randomized clinical trials, systematic reviews, and meta-analyses investigating the association between nutritional interventions and AD published between 2018 and 2022 in the PubMed, Web of Science, Scopus, and Cochrane Library databases. A total of 38 studies were identified, of which 17 were randomized clinical trials, and 21 were systematic reviews and/or meta-analyses.

**Results:**

The results show that the western diet pattern is a risk factor for developing AD. In contrast, the Mediterranean diet, ketogenic diet, and supplementation with omega-3 fatty acids and probiotics are protective factors. This effect is significant only in cases of mild-to-moderate AD.

**Conclusion:**

Certain nutritional interventions may slow the progression of AD and improve cognitive function and quality of life. Further research is required to draw more definitive conclusions.

## Introduction

Alzheimer’s disease (AD) is a progressive and irreversible neurodegenerative disease characterized by decline in cognitive and functional abilities, episodic loss of memory and language skills, neuropsychiatric symptoms, and premature death. Currently, dementia affects approximately 25 million people worldwide, and it is estimated that due to the increase in life expectancy by the year 2050, there will be at least 115.4 million people with this disease. Within the group of all dementias, it is estimated that it occupies a frequency between 60 and 80%, and is thus being the most common form of dementia. Although the cause of AD is not well-understood, is manifests at the biochemical level, it manifests as the accumulation of amyloid-beta peptide (Aβ) deposits and the formation of neurofibrillary tangles of tau protein in the brain. Currently, there is no effective treatment to reverse this situation. In addition, pharmacological treatments usually have adverse effects that can worsen patient. Research carried out in animal models shows that there is a relationship between nutrition and the biochemistry of Alzheimer’s disease. However, the knowledge of the exact mechanisms is still scarce. It has been shown that adopting certain measures can slow down its progression, among which the nutritional approach has become increasingly important (Agahi et al., 2018; Samadi et al., 2019; Yilmaz and Arica Polat, 2019; Goncalves Tosatti et al., 2022; Shrestha et al., 2022; Simsek and Uçar, 2022; Miculas et al., 2023).

Recent findings suggest that AD onset and development are strongly correlated with lifestyle, including diet. Appropriate nutritional intervention may be a good approach for delaying neurocognitive decline and reducing the risk of AD onset and development. Following a healthy dietary pattern, a high intake of plant-based foods, probiotics, nuts, and omega-3 polyunsaturated fatty acids and a low intake of saturated fats, animal-based proteins, and refined sugars can decrease the risk of neurocognitive impairment (Pistollato et al., 2018; Abduljawad et al., 2022).

In addition, vitamins and minerals serve numerous vital functions, including modulation of brain health and cognitive function. Therefore, administering these micronutrient supplements can help maintain adequate cognitive activity or even prevent dementia (Karthika et al., 2022). However, the evidence in this field of research is still limited (Rutjes et al., 2018). Therefore, the objective of this review was to identify and map the updates of the last 5 years regarding the nutritional status and nutritional interventions associated with AD patients.

## Materials and methods

### Study design

Systematic review.

### Search strategy and data sources

Between December 2022 and January 2023, a search was carried out for documents published in the last 5 years in the PubMed, Web of Science, Scopus, and Cochrane Library databases. The keywords used to search for articles were: Alzheimer Disease, Alzheimer Dementia, Alzheimer’s Disease, Antioxidant, Caloric restriction, Carotenoids, Choline, DHA, Diet, Diet intervention, Dietary pattern, Docosahexaenoic, Eicosapentaenoic, Fatty acids, Fish oil, Green tea, Ketonic diet, Mediterranean diet, Microbiota, Micronutrient, Nutrient, Nutrition, Oil, Olive oil, Omega-3, Polyphenol, Prebiotic, Probiotic, PUFA and Resveratrol. The Boolean operator used were AND and OR. The exact search equation can be found in the Supplementary material.

### Inclusion criteria

The selected documents were (1) randomized clinical trials, systematic reviews, and meta-analyses that explored the relationship between nutrition and Alzheimer’s disease, (2) published between 2018 and 2022, (3) in English; (4) implemented in the over-18 years-old population, men, and/or women, and (5) full text available.

### Exclusion criteria

The exclusion criteria were (1) studies that were not performed in humans and (2) studies that were not related to the topic of this review, such as other types of dementias or pharmacological interventions. Two researchers searched and screened the documents, and the consensus of the researchers resolved discrepancies regarding the selected documents.

## Results

### Study characteristics

**FIGURE 1:**
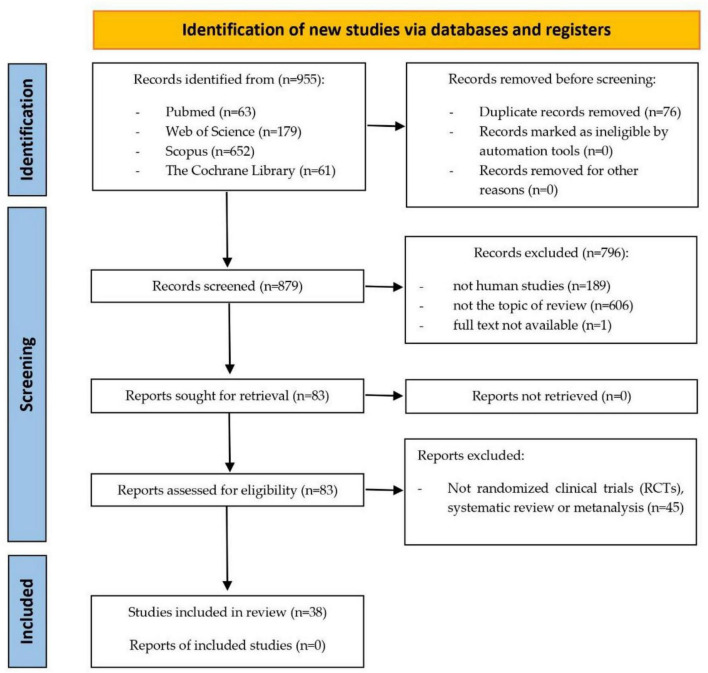
Flow chart.

A total of 955 documents were found, of which 38 articles were included in the study. Figure 1 summarizes the selection process of the studies included in this review. Regarding the epidemiological design of the included studies, 17 studies were randomized clinical trials (RCT), and 21 were systematic reviews and/or meta-analyses. Table 1 describes the main characteristics of the studies included in this review.

**TABLE 1:** Main characteristics of the studies included in this review.

References	Objectives	Method/Sample/Duration	Measuring instruments	Intervention design	Results
Doorduijn et al. (2019)	To examine potential difference in energy and protein intake in patients with MCI and AD compared to controls as a possible mechanism for unintended weight loss.	Systematic review and meta-analysis *n* = 7 articles	Food diary, 24 h recall, survey of food intake	Case-control design (patients with AD and control)	No lower energy and protein intake of patients with AD compared to controls.
Wu et al. (2022)	To study the effect of nutritional support under the clinical nursing path on the nursing effect, quality of life, and nutritional status of elderly patients with AD.	RCT *n* = 110 patients	Nursing efficiency, QLI, PSQI, MSSNS, SDSS, MNA	Nutritional support (*n* = 55) control group (*n* = 55)	Nutritional support was associated with significantly QLI scores and significant lower PSQI, MSSNS, and SDSS scores, and fewer malnourished cases versus routine nursing (*p* < 0.05).
García-Casares et al. (2021)	To conduct a systematic review and meta-analysis to determine the effects of a higher adherence to MD on MCI and AD.	Systematic review and meta-analysis *n* = 11 articles	MDA, FFQ	OS (cross-sectional, case-control or longitudinal cohort studies) and RCT	Higher adherence to MD was associated with a significantly lower risk of MCI and lower risk of AD.
Hill et al. (2019)	To summarize the evidence relating diet and nutrition to the hallmark AD biomarkers (tau and β-amyloid).	Systematic review and meta-analysis *n* = 5 articles	Glycemic indices, MDA, PET imaging, CSF levels, plasma biomarkers of AD, αβ- amyloid and tau	RCT, cross sectional and longitudinal studies	Significant effect (*p* = 0.002) of diet on AD biomarkers. Adherence to a MeDi-styled dietary pattern was associated with a reduction in AD biomarkers and subsequent pathology. Adherence to a high-glycemic, high saturated fat diet was associated with an increase in AD biomarker burden.
Samadi et al. (2019)	To review the evidence on the relation between dietary pattern and AD.	Systematic review *n* = 26 articles	FFQ, food record	OS (prospective, retrospective, cross-sectional or case-control studies)	Adherence to healthy diet can decrease oxidative stress and inflammation and accumulation of amyloid-β and consequently can decrease the risk of AD.
Hoscheidt et al. (2022)	To compare the effects of the diet intervention on metabolic parameters, CSF biomarkers, cerebral perfusion assessed with MRI, and cognition.	RCT *n* = 87 participants 4 weeks	Lumbar puncture, blood biomarkers, cognitive tests, MRI	Med-diet (*n* = 44) West-diet (*n* = 43)	Dietary patterns are powerful modulators of metabolic function, cerebrovascular health, AD pathology, and cognitive function.
Brandt et al. (2019)	To establish the feasibility of implementing the MAD in older adults with early AD or MCI due to AD. To examine whether changes in participants′ cognition, behavioral, and emotional functioning are more favorable in those in the MAD than in those in a control diet.	RCT parallel, controlled trial *n* = 14 patients 12 weeks	MMSE-2-EV, POMS-Bi	MAD (*n* = 9) Control (*n* = 5)	The MAD may not be very easy to implement in the MCI/mild AD population. It may be useful for patients that agree to continue this diet.
de la Rubia Orti et al. (2018)	To detect changes in the main cognitive functions of patients with AD after following a coconut oil enriched MD, and to determine whether there are differences in function of stage or sex.	RCT *n* = 44 patients 21 days	7 min screen	Coconut oil enriched Mediterranean Diet (*n* = 22) Control (*n* = 22)	After intervention with coconut oil, improvement in episodic, temporal orientation, and semantic memory were observed, and it seems that the positive effect is more evident in women with mild-moderate state, although other improvements in males and severe state were also shown.
Phillips et al. (2021)	To determine whether a 12 weeks modified ketogenic diet was well-tolerated and improved cognition, daily function, or quality of life in a hospital clinic of AD patients	RCT crossover *n* = 26 patients 12 weeks	ACE-III, ADCS-ADL, QLI	Modified ketogenic diet (*n* = 13) usual-ketogenic diet (*n* = 13)	Compared with a usual diet supplemented with low-fat healthy-eating guidelines, patients on the ketogenic diet improved in daily function and quality of life.
Lin et al. (2022)	To determine whether the n-3 PUFAs supplementation in different regimens could modulate the levels of pro-inflammatory cytokines and restore some cognitive and functional abilities, as well as mood status in patients with cognitive impairment.	RCT placebo-controlled 163 MCI or AD patients 24 months	Cognitive and functional abilities, biochemical, and inflammatory cytokines profiles	Placebo (*n* = 40) DHA (*n* = 41) EPA (*n* = 40) DHA + EPA (*n* = 42)	n-3 PUFAs supplements did not reduce cognitive, functional, and depressive symptom outcomes, but spoken language ability and constructional praxis subitems of ADAS-cog did.
Jernerén et al. (2019)	To investigate whether baseline levels of plasma tHcy, a marker of B vitamin status, modify the effects of n-3-PUFAs supplementation on cognitive performance in moderate AD.	RCT 171 patients 6 months	Plasma aminoacids, MMSE, ADAS-cog, Global CDR, CDRsob	n-3 PUFAs (*n* = 88) placebo (*n* = 83)	The effect of n-3 PUFAs supplementation on MMSE and CDR appears to be influenced by baseline tHcy, suggesting that adequate B vitamin status is required to obtain beneficial effects of n-3 PUFAs on cognition.
Nolan et al. (2022)	To build on the existing exploratory research and investigate the impact of the micronutrients on the natural progression of AD in a RCT.	RCT *n* = 77 patients 12 months	Blood analysis, MMSE, CDR	Fish oil, carotenoids, vitamin E (*n* = 50) placebo (*n* = 27)	The active group also performed better in objective measures of AD severity (i.e., memory and mood), with a statistically significant difference reported in the clinical collateral for memory (*p* < 0.001).
Albrahim (2020)	To find out the potential role of nutritional components in improving brain function among patients with AD.	Systematic review and meta-analysis *n* = 19 RCT	Omega-3, DHA, vitamin C, E, B12, folate, homocysteine	RCT	Chain-free SFA and TFA occur in greater amounts in the brains of individuals with AD than in those without AD.
Araya-Quintanilla et al. (2020)	To determine if there is scientific evidence of the effectiveness of omega-3 supplementation in improving cognitive function in patients with AD.	Systematic review and meta-analysis *n* = 6 articles	MMSE, ADAS-COG, CDR, NPI	RCT	No consistent evidence to support the effectiveness of omega-3 supplementation in improving cognitive function in AD patients in the short and medium term.
Zhu et al. (2021)	To provide new evidence on relationships between dietary fatty acid intake and cognition.	Prospective cohort meta-analysis *n* = 14 articles	Dietary fatty acids intake	Prospective cohort study	The intake of total fatty acids, SFAs, MUFAs, PUFAs, and omega-3 PUFAs was not significantly associated with dementia risk. However, omega-3 PUFA intake may be negatively associated with MCI risk.
Canhada et al. (2018)	To evaluate the effects of omega-3 fatty acids supplementation on cognitive function in AD patients.	Systematic review *n* = 7 articles	ADAS-cog, CIBIC-plus, MMSE, HDRS, MADRS, CDR, ADCS-ADL, ADCS-IADL, CGB, DAD, NPI, NTB, BADLS	RCT	Omega-3 fatty acids may be beneficial in disease onset, when there is slight impairment of brain function.
Moreira et al. (2020)	To evaluate the effect of dietary interventions on the cognitive performance of individuals with AD.	Systematic review *n* = 32 RCT	MMSE, ADAS-cog, CDR-SOB	RCT	Omega-3 fatty acid showed positive effects at different doses. Probiotic, ginseng, inositol and specialized nutritional formulas seemed to have a positive effect on cognition.
Samieri et al. (2018)	To conduct a statistically powerful investigation of fish intake and decline in global cognition and episodic memory.	Systematic review *n* = 5 articles	FFQ, global cognition, episodic memory	Cohort studies	Higher fish intake was associated with slower decline in both global cognition and memory (*p* < 0.031).
Agahi et al. (2018)	To evaluate responsiveness of the inflammatory and oxidative biomarkers to the probiotic treatment.	RCT double blind 48 patients 12 weeks	Test Your Memory, serum concentrations	Probiotic (*n* = 25) control (*n* = 23)	Cognitive and biochemical indications in the patients with severe AD are insensitive to the probiotic supplementation.
Akbari et al. (2020)	To assess the effects of probiotic supplementation on cognitive function and metabolic status.	RCT double-blind controlled trial *n* = 60 patients	MMSE	Probiotics (*n* = 30) control (*n* = 30)	The probiotic treated patients showed a significant improvement in the MMSE score (*p* < 0.001).
Doulberis et al. (2021)	To investigate the accessible regarding possible association between AD and gastrointestinal microbiota.	Systematic review *n* = 24 articles	Gut microbiota analysis	OS	Positive association between gastrointestinal microbiota and deterioration of AD.
González Cordero et al. (2022)	To provide the best scientific evidence available on the relationship between the gut microbiota and AD.	Systematic review *n* = 8 articles	Gut microbiota analysis	OS	There is a high association between the decrease in the richness of the microbiota and the incidence of AD.
Liu et al. (2022)	To evaluate the clinical value of intestinal flora balance therapy supported by probiotics in improving cognitive function and symptoms in patients with AD.	Systematic review and meta-analysis *n* = 5 RCT	MMSE, instant memory score, ADAS-cog, ADL	RCT	Intestinal flora balance therapy based on probiotic support can effectively improve cognitive function, instantaneous memory, and ability of daily life in patients with AD.
Tamtaji et al. (2019)	To determine the effects of probiotic and selenium supplementation on cognitive function and metabolic status among patients with AD.	RCT double blind controlled trial 79 patients 12 weeks	MMSE, biochemical analysis, gene expression	Selenium + probiotics (*n* = 27) selenium (*n* = 26) placebo (*n* = 26)	Probiotic and selenium supplementation improved cognitive function and biochemical profiles.
Mullan et al. (2018)	To compare the plasma antioxidant status of individuals with AD and cognitively intact controls.	Meta-analysis of case-control studies *n* = 52 articles	α-carotene, β-carotene, lycopene, β-cryptoxanthin, lutein, zeaxanthin, vitamin A, C, E, uric acid	Case-control studies	Patients with AD had significantly lower plasma levels of α-carotene, β-carotene, lycopene, lutein, vitamin A, C, and E, and uric acid.
Qu et al. (2021)	To evaluate the associations of six major members of carotenoids with the occurrence of AD by conducting systematic review and meta-analysis.	Systematic review and meta-analysis *n* = 16 articles	Plasma and serum carotenoids analysis	Cross-sectional study, cohort study, case-control study	Lutein and zeaxanthin concentrations in plasma/serum were inversely related to the risk of AD.
Chen et al. (2021)	To evaluate the combined action of folic acid and vitamin B12 supplementation on cognitive performance and inflammation in patients with AD.	RCT single-blinded placebo-controlled trial 120 patients 6 months	Cognitive performance, blood folate, vitamin B12, inflammatory cytokine levels	Folic acid + vitamin B12 (*n* = 60) placebo (*n* = 60)	Folic acid and vitamin B12 supplementation showed a positive therapeutic effect in AD patients who were not on a folic acid-fortified diet.
Chai et al. (2019)	To comprehensively explore the associations between serum 25(OH)D deficiency and risk of dementia and AD.	Meta-analysis *n* = 12 articles	Vitamin D concentration	Prospective cohort, cross-sectional	There are significant associations between vitamin D deficiency and both dementia and AD. There are stronger associations between severe vitamin D deficiency (<10 ng/ml) and both dementia and AD compared to moderate vitamin D deficiency (10–20 ng/ml).
Jayedi et al. (2019)	To test the dose-response association of serum 25(OH)D and risk of dementia and AD.	Meta-analysis *n* = 8 cohort studies	Vitamin D concentration	Prospective cohort, retrospective cohort	Higher levels of serum 25(OH)D were associated with a lower risk of dementia and AD.
Du et al. (2020)	To investigate if vitamin D supplementation can prevent AD.	Systematic review and meta-analysis of RCT *n* = 9 articles	MMSE	RCT	No significant difference in the MMSE, verbal fluency, verbal memory, visual ability, and attention scores between the vitamin D group and comparison group.
Yang et al. (2019)	To synthesize the association of serum vitamin D concentrations with AD in adults.	Meta-analysis of prospective cohort studies *n* = 6 articles	Vitamin D concentration	Prospective cohort	Serum vitamin D deficiency (<25 nmol/L) or insufficiency (25–50 nmol/L) was not statistically significant and associated with the risk of AD.
Jia et al. (2019)	To assess the effect of a 12 months vitamin D supplementation on cognitive function and amyloid β-related biomarkers in subjects with AD.	RCT double-blind placebo-controlled 210 patients 12 months	Test of cognitive performance and αβ-related biomarkers	Vitamin D (*n* = 105) placebo (*n* = 105)	Vitamin D group had significant increase in full scale IQ during follow-up period (*p* < 0.001).
Belitskaya-Lévy et al. (2018)	To examine the role of APOE genotypes on the effect of treatment in delaying the rate of functional decline of AD.	RCT *n* = 415 6 months	ADCL-ADL	ε4 non-carriers (*n* = 209) ε4 carriers (*n* = 206)	Vitamin E group had slower functional decline than those receiving placebo.
Buglio et al. (2022)	To assess the effects of resveratrol on MCI and AD.	Systematic review *n* = 5 articles	Brain volume, MMSE	Interventional studies	In AD patients, the use of resveratrol improves brain volume, reduces the MMSE, and improves AD scores. In patients with MCI, this polyphenol prevents decline in Standard Volumes of Interest and increases the Resting-state Functional Connectivity score.
Fang et al. (2022)	To explore the effect of resveratrol combined with donepezil hydrochloride on inflammatory factor level and cognitive function level of patients with AD.	RCT *n* = 90 patients 12 months	MMSE, FIM, ADAS-cog	Resveratrol (*n* = 45) control (*n* = 45)	Resveratrol group obtained significantly higher good rate, MMSE score, and FIM score (*p* < 0.05) and lower clinical indicators and ADAS-cog score (*p* < 0.001).
Gu et al. (2021)	To investigate the antagonistic effect of trans-resveratrol on moderate to mild AD.	RCT *n* = 30 patients 52 weeks	MRI, CSF	Trans-resveratrol (*n* = 15) placebo (*n* = 15)	Neuroprotective role of trans-resveratrol in patients with mild to moderate AD.
Zhu et al. (2018)	To evaluate the safety, tolerability, and efficacy of an oral preparation of resveratrol, glucose, and malate (RGM) in slowing the progression of AD.	RCT double-blind placebo-controlled *n* = 39 patients 12 months	ADAS-cog, CIBIC-plus, MMSE, ADCS-ADL, NPI	Resveratrol (*n* = 17) placebo (*n* = 15)	Change scores on ADAS-cog, MMSE, ADCS-ADL, or NPI all showed less deterioration in the treatment than the control group; however, none of the change scores was significant.
Kakutani et al. (2019)	To examine the association between green tea intake and dementia, AD, MCI, or cognitive impairment.	Systematic review *n* = 8 articles	Food intake, FFQ	OS	Green tea intake might reduce the risk of dementia, AD, MCI, or cognitive impairment.

ACE-III, addenbrookes cognitive examination-III scale; AD, Alzheimer’s disease; ADAS-cog, Alzheimer’s disease assessment scale-cognitive section; ADCS-ADL, Alzheimer’s disease cooperative study-activities of daily living; ADCS-IADL, Alzheimer’s disease cooperative study-instrumental activities of daily living; BADLS, Bristol’s activities of daily living scale; CDR, clinical dementia rating scale; CDR-SOB, clinical dementia rating scale-sum of boxes; CGB, caregivers burden scale; CIBIC-plus, clinicians global impression of change; CSF, cerebrospinal fluid; DAD, disability assessment for dementia scale; FFQ, food frequency questionnaire; HDRS, Hamilton depression scale; MAD, modified Atkins diet; MADRS, Montgomery Asberg depression rating scale; MCI, mild cognitive impairment; MD, Mediterranean diet; MDA, Mediterranean diet adherence; MMSE, mini-mental state examination; MNA, mini nutritional assessment; MRI, magnetic resonance imaging; MSSNS, mental status scale in non-psychiatric settings; NPI, neuropsychiatric inventory; NTB, neuropsychological test battery; tHcy, total homocysteine; OS, observational studies; PSQI, Pittsburgh Sleep Quality Index; QLI, quality of life index; RCT, randomized controlled trial; SDSS, social disability screening schedule.

### Role of diet in Alzheimer’s disease

Protein-calorie malnutrition is strongly correlated with patients with cognitive decline and AD (Doorduijn et al., 2019). Wu et al. (2022) found that adequate nutritional support can significantly improve the quality of life, cognitive function, and psychological and nutritional status of elderly AD patients. Furthermore, nutritional support was associated with better sleep quality as measured by the Pittsburgh Sleep Quality Index (PSQI) (Wu et al., 2022).

An unhealthy diet pattern, such as a high-fat diet with a high glycemic load and high cholesterol or a Western diet, is an important risk factor for neurodegeneration because it increases Aβ peptide stores and other biomarkers of neurodegeneration in AD. Conversely, following a healthy dietary pattern, such as a DASH (Dietary Approaches to Stop Hypertension), Mediterranean, or low-fat diet, has neuroprotective effects in preventing AD. The main mechanisms are based on the reduction of oxidative stress and inflammation and a lower accumulation of Aβ peptides. Greater adherence to the Mediterranean diet decreased the levels of IL-6, TNF-α, CRP, and LDL. The DASH diet is characterized by low consumption of red and processed meat and high consumption of fruits, vegetables, and whole grains. The nutritional profile is high in potassium, calcium, magnesium, and fiber, whereas the sodium and saturated fat contents are relatively low. For this reason, both the Mediterranean and DASH diets have anti-inflammatory effects and are capable of reducing oxidative stress, exerting a protective role against AD (Hill et al., 2019; Samadi et al., 2019; García-Casares et al., 2021). In addition, the Mediterranean diet provides numerous health benefits, including improved cerebral perfusion. However, this effect is more significant in patients with AD in the mild or early stages, whereas the effect in more advanced stages is not noticeable. In contrast, the Western dietary pattern increases the risk of AD by altering metabolic health and reducing cerebral perfusion and thus impairing cognition (Hoscheidt et al., 2022).

Patients with AD present with alterations in brain metabolism. Recent studies have argued that ketone bodies can help correct this situation. Ketone bodies are a direct source of cellular energy from fat metabolism and can be used as a source of energy for the brain when the glucose supply is limited. Recent research suggests that ketone bodies may improve episodic memory, temporary memory, semantic memory, and vitality in patients with early AD, and this effect is more evident in women. Ketone bodies have also been shown to improve functional capacity and quality of life, which are essential for AD patients. However, one of the most common problems with the ketogenic diet is that it is difficult to maintain over the long term because of its characteristics. Phillips et al. (2021) stated that high rates of retention, adherence, and safety can be achieved when it is well-established (de la Rubia Orti et al., 2018; Brandt et al., 2019).

### Effect of fatty acids in Alzheimer’s disease

The use of omega-3 fatty acid in AD has been widely studied. Nevertheless, omega-3 fatty acids did not improve cognitive and functional decline nor depressive symptoms in an RCT conducted by Lin et al. (2022) but spoken language ability did. It seems that this biomolecule must interact with other micronutrients to achieve this effect. Jernerén et al. (2019) suggested that adequate levels of B vitamins are needed for omega-3 fatty acids to effect cognition. On the other hand, other micronutrients, such as docosahexaenoic acid (DHA), eicosapentaenoic acid (EPA), carotenoids, or vitamin E, can also help improve memory and mood. This effect is significant only in milder-to-moderate stages of the disease (Nolan et al., 2022).

Patients with AD tend to have higher serum saturated and trans-fatty acid concentrations than the general population. This is associated with alterations in the metabolomics of the tyrosine, tryptophan, purine, and tocopherol pathways. Albrahim’s meta-analysis found that supplementation with these nutrients can improve cognitive decline, functional connectivity, and brain atrophy (Albrahim, 2020). We found more controversy regarding the effects of omega-3 fatty acids. Meta-analyses performed by Zhu et al. and Araya-Quintanilla et al. found no decreased risk of dementia or improved cognitive function with supplementation of these fatty acids (Araya-Quintanilla et al., 2020; Zhu et al., 2021). Other studies have indicated that omega-3 fatty acids can delay cognitive aging and memory decline; however, this effect is only demonstrable in cases of mild-to-moderate dementia or when brain function decline is still ongoing in the earliest stages of the disease (Canhada et al., 2018; Samieri et al., 2018; Moreira et al., 2020).

### Microbiota-brain axis

The study of microbiota-health interactions has gained great importance in the last century. The composition of microbiota affects the development and evolution of numerous diseases, including AD. AD patients have been shown to have an altered microbiota, which promotes a pro-inflammatory state, affects cognitive function, and increases the risk of neurodegeneration through the gut-brain axis. Therefore, probiotic supplementation may improve metabolic abnormalities and attenuates inflammation and oxidative stress. Additionally, it can improve cognitive function. However, these effects depend on the formulation and dose of the probiotic bacterium, severity of the disease, and timing of probiotic administration. On the other hand, a deficit of certain nutrients, such as omega-3 PUFAs, decreases the resistance to neurotoxicity produced by this altered microbiota and affects the central nervous system. Therefore, in addition to correcting the altered microbiota with probiotics and prebiotics, it is necessary to correct the deficit of the nutrients that interact with the microbiota. Correction of altered gut microbiota using probiotics may improve cognitive function and instant memory in patients with AD (Agahi et al., 2018; Tamtaji et al., 2019; Akbari et al., 2020; Doulberis et al., 2021; González Cordero et al., 2022; Liu et al., 2022).

### Vitamins and other antioxidants related to Alzheimer’s disease

Vitamins play an important role in the pathogenesis of AD. Patients are observed to have lower plasma levels of α-carotene, β-carotene, lycopene, lutein, vitamins A, C, and E, and uric acid than the general population (Mullan et al., 2018; Qu et al., 2021). An RCT by Chen et al. (2021) found that combined folic acid and vitamin B_12_ supplementation significantly improved cognitive performance and inflammation in patients with AD. Vitamin D, it is observed that vitamin D deficiency is significantly associated with the development of dementia and AD. The stronger the association, the greater the vitamin D deficiency (<10 ng/ml). Conversely, when serum vitamin D concentrations increase, the risk of dementia decreases (Chai et al., 2019; Jayedi et al., 2019). However, other studies, such as those by Yang et al. (2019), Du et al. (2020) found no significant association between vitamin D deficiency and improvements in AD cognitive parameters. The RCT by Jia et al. (2019) found that vitamin D supplementation for at least 12 months significantly improved cognitive function and Aβ peptide-related biomarkers. The apolipoprotein E(APOE) genotype is a known risk factor for AD. Vitamin E administration may help delay cognitive decline by modulating the response to treatment in APOE genotypes (Belitskaya-Lévy et al., 2018).

It is also suggested that resveratrol supplementation may improve cerebrovascular function and reduce the risk of developing dementia. The mechanism could be due to the fact that resveratrol is able to activate Sirt-1 and inhibit COX-2, 5-lipoxygenase and NFkB, resulting in less activation of proinflammatory pathways (Buglio et al., 2022). Other studies suggested that this is related to the Aβ peptide, which accumulates in the brains of people with AD. Resveratrol can act as an AD antagonist, fulfilling neuroprotective functions, improving inflammation levels, and promoting cognitive functions. The mechanism involves a reduction in Aβ accumulation and toxicity in the brain of these patients and a reduction in neuroinflammation. The neuroprotective effect has only been found to be significant in patients who are in the early stages of the disease (Zhu et al., 2018; Gu et al., 2021; Fang et al., 2022). In contrast, a systematic review by Kakutani et al. found that green tea intake can reduce the risk of dementia, AD, and general cognitive decline (Kakutani et al., 2019).

## Discussion

Low dietary quality is a risk factor for the development AD, thus, worsening cognitive performance and verbal fluency (Hossain et al., 2019). Additionally, malnutrition and unintentional weight loss are associated with an increased risk of mortality in patients with AD (de Sousa et al., 2020). Consumption of refined carbohydrates or a diet with a high glycemic index is associated with increased accumulation of Aβ peptides in the brain. This effect is even worse in APOE-ε4 carriers, which is a genetic risk factor associated with AD and dementia, as well as insulin resistance. However, the exact mechanisms underlying this relationship remains unknown (Gentreau et al., 2020; Taylor et al., 2021). A Western diet pattern increases the risk of AD, as this diet increases inflammation levels (Wieckowska-Gacek et al., 2021). On the contrary, according to the Spain Dementia Cohort, adherence to the Mediterranean diet is associated with a 20% lower risk of dementia. The Mediterranean diet has been shown to improve cognitive outcomes, increase gray matter volume, improve memory, and decrease memory decline (Nutaitis et al., 2019; Ballarini et al., 2021; Encarnacion Andreu-Reinon et al., 2021). A ketogenic diet may also be useful in the treatment of AD, as it has been shown to reduce oxidative stress and inflammation and reduce the negative effects of altered glucose metabolism in the brain. In addition, according to other clinical studies, this diet can improve verbal memory, attention, and overall cognitive function. However, long-term use of this diet may present risks; therefore, it should be monitored by an expert nutritionist (Simsek and Uçar, 2022).

Because AD presents with high levels of oxidative stress, adequate intake of antioxidants in the diet is a factor to be consider considered. Whether oxidative status is a cause or product of AD remains unknown (Socha et al., 2021); however, vitamin intake has been reported to help combat cognitive and memory decline (Alam, 2022). Lower levels of vitamin D are associated with worse cognitive performance scores in patients (Yilmaz and Arica Polat, 2019). However, according to the results of our review, the supplementation of vitamin D on improving AD state still lacks enough evidence (Chai et al., 2019; Jayedi et al., 2019; Jia et al., 2019; Yang et al., 2019; Du et al., 2020). Vitamin B_12_ deficiency is a fairly frequent condition in the elderly population and is a risk factor for AD (Shrestha et al., 2022). Vitamin E is a powerful antioxidant and an anti-inflammatory agent. Observational cohort studies have shown that compared to the general population, people with AD have significantly lower levels of tocopherols, tocotrienols, and total vitamin E (Casati et al., 2020). The same is true for choline (Yuan et al., 2022). On the contrary, we did not find any study that met the inclusion criteria for the nutritional intervention with vitamin C in patients with AD, which could be an interesting topic for future research. The studies included in our review indicated that the effects of nutritional interventions only work in patients with mild and moderate AD (Hoscheidt et al., 2022; Nolan et al., 2022). We did not find articles explaining why they are not useful in severe AD patients, although we think that a possible cause could be related to the high level of oxidative stress. Even though, studies with high quality done in humans about supplementation of vitamins on this topic are limited.

The study of fatty acids as nutritional factors for dementia is also a popular research topic. Adequate levels of omega-3 polyunsaturated fatty acids, especially EPA and DHA, are associated with slower rates of cognitive decline and reduced risk of AD. Therefore, it is important to advise AD patients to include fish, nuts, seeds, and vegetable oils in their diet (Gustafson et al., 2020; Chu et al., 2022; Li et al., 2022). However, the significant efficacy is still unknown, as demonstrated by our results (Lin et al., 2022). The microbiota, it is known to be a factor that must be considered in AD, as dysbiosis is a clear risk factor for the development of AD. This is because of the metabolites produced by the microbiota, which can modulate the biochemical state of the brain. This connection is called the “gut-brain axis.” In addition, high-fat diets, the use of antibiotics, or the lack of probiotics and/or prebiotics can also change the composition of the microbiota and therefore be a risk factor for AD (Bello-Corral et al., 2021; Khedr et al., 2022; Szablewski, 2022). However, in this study, we found that AD was correlated with high levels of inflammation and oxidation (Goncalves Tosatti et al., 2022; Gu et al., 2022), which should be considered in future research and clinical treatments.

Most of the points included in our review are similar to those of other articles related to this field; thus, it seems that nutrition can protect and/or decrease the progression rate of AD. On the other hand, during the bibliographic search, a large number of articles investigated the relationship between microbiota and AD and malnutrition and AD, which suggests that these research topics have been topics of interest in recent years, since we filtered for the last 5 years. We also found a high percentage of studies carried out in postmortem humans, that evaluated the biochemical composition of the brain. This is one of the limitations of the study of AD, since the psychological and cognitive state is often not related to the physical and biochemical state of the brain. This is one of the reasons for the difficulty in conducting RCT with good methodological quality to study the relationship between nutrition and AD (Liu et al., 2022). Nutritional interventions are good non-pharmacological tools for the treatment of AD. However, more studies with effective methodological quality are needed to draw better conclusions.

## Conclusion

The results showed that nutritional interventions are capable of slowing down the rate of progression of Alzheimer’s disease, improving cognitive function, and improving the quality of life of these patients. However, many knowledge gaps remain to be investigated; therefore, a deeper study on the association between nutrition and AD is recommended.

## Author contributions

All authors listed have made a substantial, direct, and intellectual contribution to the work, and approved it for publication.
