# Supplementary material for: Effect of nutrition in Alzheimer’s disease: A systematic review
Source: Front Neurosci. 2023 May 4;17:1147177. doi: 10.3389/fnins.2023.1147177 (PMC10194838; doi:10.3389/fnins.2023.1147177)
Supplement: Supplementary file 1 [file Data_Sheet_1.docx]

**Supplementary material**

Search equation used in our review

(“Alzheimer Disease” OR “Alzheimer Dementia” OR “Alzheimer's Disease”) AND (“Antioxidant” OR “Caloric restriction” OR “Carotenoids” OR “Choline” OR “DHA” OR “Diet” OR “Diet intervention” OR “Dietary pattern” OR “Docosahexaenoic” OR “Eicosapentaenoic” OR “Fatty acids” OR “Fish oil” OR “Green tea” OR “Ketonic diet” OR “Mediterranean diet” OR “Microbiota” OR “Micronutrient” OR “Nutrient” OR “Nutrition” OR “Oil” OR “Olive oil” OR “Omega-3” OR “Polyphenol” OR “Prebiotic” OR “Probiotic” OR “PUFA” OR “Resveratrol”)
